# Transcriptomic Analysis of mRNA-lncRNA-miRNA Interactions in Hepatocellular Carcinoma

**DOI:** 10.1038/s41598-019-52559-x

**Published:** 2019-11-06

**Authors:** Xia Tang, Delong Feng, Min Li, Jinxue Zhou, Xiaoyuan Li, Dachun Zhao, Bingtao Hao, Dewei Li, Keyue Ding

**Affiliations:** 1 Key Laboratory of Molecular Biology for Infectious Diseases (Ministry of Education), Institute for Viral Hepatitis, Department of Infectious Diseases, The Second Affiliated Hospital, Chongqing Medical University, Chongqing, P.R. China; 2 Cancer Research Institute, Southern Medical University, Guangzhou, Guangdong P.R. China; 3 Department of Hepatobiliary Surgery, Suining Central Hospital, Suining, Sichuan Province P.R. China; 4 Department of Hepatobiliary Surgery, Henan Tumor Hospital, Zhengzhou, Henan P.R. China; 5 Department of Medical Oncology, Peking Union Medical College Hospital, Peking Union Medical College and Chinese Academy of Medical Sciences, Beijing, P.R. China; 6 Department of Pathology, Peking Union Medical College Hospital, Peking Union Medical College and Chinese Academy of Medical Sciences, Beijing, P.R. China; 7 Henan Medical Genetics Institute, People’s Hospital of Henan University, Zhengzhou, Henan P.R. China; 8 Department of Hepatobiliary Surgery, The First Affiliated Hospital of Chongqing Medical University, Chongqing, P.R. China; 9 Department of Bioinformatics, School of Basic Medicine, Chongqing Medical University, Chongqing, P.R. China

**Keywords:** Cancer epigenetics, Hepatocellular carcinoma

## Abstract

Fully elucidating the molecular mechanisms of non-coding RNAs (ncRNAs), including micro RNAs (miRNAs) and long non-coding RNAs (lncRNAs), underlying hepatocarcinogenesis is challenging. We characterized the expression profiles of ncRNAs and constructed a regulatory mRNA-lncRNA-miRNA (MLMI) network based on transcriptome sequencing (RNA-seq) of hepatocellular carcinoma (HCC, *n* = 9) patients. Of the identified miRNAs (*n* = 203) and lncRNAs (*n* = 1,090), we found 16 significantly differentially expressed (DE) miRNAs and three DE lncRNAs. The DE RNAs were highly enriched in 21 functional pathways implicated in HCC (*p* < 0.05), including p53, MAPK, and NAFLD signaling. Potential pairwise interactions between DE ncRNAs and mRNAs were fully characterized using *in silico* prediction and experimentally-validated evidence. We for the first time constructed a MLMI network of reciprocal interactions for 16 miRNAs, three lncRNAs, and 253 mRNAs in HCC. The predominant role of *MEG3* in the MLMI network was validated by its overexpression *in vitro* that the expression levels of a proportion of *MEG3*-targeted miRNAs and mRNAs was changed significantly. Our results suggested that the comprehensive MLMI network synergistically modulated carcinogenesis, and the crosstalk of the network provides a new avenue to accurately describe the molecular mechanisms of hepatocarcinogenesis.

## Introduction

Hepatocellular carcinoma (HCC) is the third cause of cancer mortality worldwide^1^. Chronic liver infection due to hepatitis B or C virus (HBV or HCV) infection is one of the most important risk factors for HCC^1^. Sequencing of multiple HCCs has characterized the landscape of genomic mutations and has identified multiple genes responsible for HCC initiation, maintenance, progression, and metastasis^2^. In addition, RNA-seq has been used to comprehensively characterize HCC transcriptomes.

Multiple molecular mechanisms are responsible for the post-transcriptional regulation and modulation of protein functions mediated by miRNAs (~22 nt) and lncRNAs (>200 nt), including partial seed-base pairing with the mRNA target, and complementary binding to the mRNA in *cis-* or *trans-*manners^3^. Dysregulation of miRNAs has been implicated in HCC via influencing apoptosis and proliferation (e.g., *let-7a*^4^), cell cycle and invasion, metastasis, and drug resistance (e.g., *miR-214*^5^). In addition, several lncRNAs have been shown to be directly involved in hepatocarcinogenesis. For example, overexpression of *HOTAIR* promotes the HCC development through interactions with *miR-1*^6^. Both miRNAs and lncRNAs may exhibit oncogenic property^7^, and may act as regulators via directly targeting mRNAs (e.g., *miR-125b-LIN2882*^8^) or acting as sponges, especially lncRNAs^9^.

The expression profiles of ncRNA in HCC have been characterized previously^2^, and interactions of miRNA-mRNA, lncRNA-mRNA, and miRNA-lncRNA have been investigated using the HCC microarray data from Gene Expression Omnibus (GEO)^10^. However, an integrated analysis of the regulatory network of HCC mediated by mRNA-lncRNA-miRNA (MLMI) network has yet to be performed. In the present study, we aimed to elucidate the co-regulatory functional MLMI network for HCC patients according to the expression profiles of DE non-coding RNAs and mRNAs based on transcriptome sequencing.

## Material and Methods

### Patients and tissue specimens

A total of nine paired HCC and adjacent non-cancerous tissues described in our previously studies^11^, were used for the RNA-seq analysis. Since the virus with reproducible potential in the serum has been cleared after antiviral treatment, the HBV DNA of four patients was negative or undetectable^12^. However, HBsAg can be expressed due to the integration of HBV into the human genome^13^, i.e., HBsAg positive. Another independent HCC cohort was used for validation (*n* = 46, Additional file 1, Table S1). This study was approved by the Institutional Review Board (IRB) of the First Affiliated Hospital of Chongqing Medical University, Suining Central Hospital, and Henan Tumor Hospital. All subjects gave written informed consent in accordance with the Declaration of Helsinki. This study was carried out in accordance with the recommendations of “the Ethnic Requirement for Human Subject Study” under each involved site. All of the experiments were performed in accordance with the relevant guidelines and regulations.

### cDNA library preparation and RNA-sequencing

Total RNAs was isolated from nine HCC and paired non-cancerous tissues using TRIzol reagent (Invitrogen Corp., Carlsbad, CA) according to the manufacturer’s protocol. The RNA purity was evaluated using the A260/A280 ratio (Thermo Fisher Scientific NanoDrop^TM^). A detailed description of cDNA library preparation for mRNA and lncRNA is provided in our previous study^11^. We used a miRNA Library Kit for library construction (QIAseq, Qiagen, Düsseldorf, Germany). The connections were sequentially connected to the 3′ and 5′ ends of the sRNAs in an unbiased reaction. Subsequently, universal cDNA synthesis, cDNA purification, library amplification and library purification were performed using unique molecular identifiers. The patented modified oligonucleotide method^14^ mostly avoided the presence of an adapter dipolymer in the sequencing library and effectively removed the major pollutants that are often observed during the sequencing process. All procedures were performed at the Beijing Genome Institute (Beijing, China).

### Bioinformatics analysis

A flowchart of the miRNA sequencing data analysis, including pre-processing and quality control (QC), identification of miRNAs and their targets, and the network construction, was shown in Fig. S1 (Additional file 1).

#### Pre-processing and QC

Raw sequencing reads with Q scores <30 or without detectable adaptors were excluded. Adaptor sequences were trimmed from the 3′ end based on an error rate of 10% (Illumina RNA adapter sequence: 5′-TGG AAT TCT CGG GTG CCA AGG-3′). We removed reads with a sequencing length <16 or >36 nt to avoid distortion and the generation of false-positive mappings. For lncRNA, we generated clean reads by removing reads containing adapter contamination and >10% poly-N, and low-quality reads (>50% of bases with Q scores ≤ 10%).

#### Identification of DE ncRNAs

We used the sRNAtoolbox implemented in sRNAbench^15^ to analyze the miRNA expression profiles. Clean reads were aligned to the human reference genome (hg19) and mature miRNAs (miRBase v.21^16^), allowing only one nucleotide mismatch. To infer DE miRNAs, we combined the use of edgeR (based on the normalized trimmed mean of the M-values (TMM) at a genome-wide significance level (FDR < 0.05))^17^, DESeq (based on ${\rm{|}}{\log }2({FC}){\rm{|}}$ > 1.2 using the normalized fragments per kilobase of transcript per million mapped reads (FPKMs)), a p-adjusted value < 0.05)^18^, which ${\log }2({FC})$ refers to the logarithm of fold change, and ‘fold change (FC)’ is the ratio of the normalized expression count (FPKM) between cancer and its paired non-cancerous sample^19^. A consensus DE miRNA was selected if it was identified by both algorithms. We used DESeq to measure the expression levels of DE miRNAs due to its superior performance^20^ and greater inclusiveness for our miRNA focus (16/17).

We re-analyzed the lncRNA expression profiles using an updated pipeline in the same cohort described previously^11^. Briefly, we mapped clean reads to the human genome (hg19) using TopHat^21^ and assembled and merged transcripts using Cufflink^22^. We combined the use of PLAR^23^ and slncky^24^ to generate plausible candidate lncRNAs. We calculated the FPKMs of the filtered lncRNAs using Cuffdiff^22^. Transcripts or genes that met the criteria of ${\rm{|}}{\log }2({FC}){\rm{|}}$ > 1.2 and FDR < 0.05 were defined as DE lncRNAs. We excluded pseudogenes or spurious lncRNAs based on manual annotation.

#### Target prediction

Regulatory relationships between any two types of RNA molecules were predicted *in silico* based on chromosomal distributions and sequence correlations, followed by evidence from previously reported (Additional file 1 Fig. S2). First, we predicted miRNA-targeted mRNAs according to potential targeted regions (e.g., the 3′ and 5′ untranslated regions (UTRs) or coding sequences (CDS) of a given mRNA) using miRWalk 2.0^25^); matched targets were retained as candidates if they were identified by at least 8 of the 12 algorithms (3′ UTR), 5 of the 6 algorithms (5′ UTR) and 5 of the 7 algorithms (CDS) (FDR < 0.05). We constructed miRNA-mRNA interactions via integration of the DE mRNAs identified in our previous study^11^ and previously reported miRNA-targeted mRNAs from miRTarBase 6.0^26^. Second, we predicted miRNA-targeted lncRNAs if they were concordantly identified using miRwalk^27^, miRanda^28^, RNAhybrid^29^, and TargetScan^30^ (FDR < 0.05), followed by previously reported evidence from starBase (v2.0)^31^. A list of HCC-related lncRNAs (*n* = 161) reported previously^29,30^ and the Lnc2Cancer database^32^ was compiled in Table S2. Finally, we searched lncRNA-targeted mRNAs via a *trans* mechanism using lncRNAtor^33^ and Co-LncRNA^34^. Therefore, we obtained a list of potential lncRNA-mRNA interactions via the combination of all lncRNA-targeted mRNAs implicated in HCC.

#### Construction of a mRNA-lncRNA-miRNA (MLMI) network

The potential interactions among RNAs indicated that a given miRNA targeted both mRNAs and lncRNAs. Additionally, miRNA-targeted lncRNAs can complementary target mRNAs. Given these reciprocal interactions, we constructed a mRNA-lncRNA-miRNA (MLMI) network that included all DE miRNAs, lncRNAs and mRNAs identified in our HCC cohort. The network robustness was measured using linkage co-expression and was calculated using WGCNA^35^.

#### Functional pathway analysis

An Over-Representation Enrichment Analysis (ORA) for potential targeted-mRNAs in KEGG, Panther, and Wiki was performed using WebGestalt^36^, assuming a minimum number of genes ≥ 5 in a given pathway. The molecular functions of the DE ncRNAs were determined using gene ontology (GO)^37^ (FDR < 0.05). We manually reviewed whether these enriched pathways were implicated in HCC.

### Survival analysis

Kaplan-Meier survival analysis was performed for the 360 HCC patients from The Cancer Genome Atlas (TCGA), who were classified into groups with a higher (upper 25%, *n* = 90) and lower (lower 25%, *n* = 90) expression levels of a given ncRNA. Statistical significance was assessed using the log-rank test (*p* < 0.05). All analyses were performed in OncoLnc^38^. It should be noted that our survival analysis was only based on molecular expression. Incorporation of liver function status such as the levels of alanine aminotransferase (ALT) and aspartate aminotransferase (AST) using multiple Cox model will model the prognosis of liver cancer more accurately.

### Validation of DE miRNAs and lncRNAs using real-time quantitative PCR

We selected one down-regulated lncRNA (MIR22HG), two up-regulated miRNAs (*miR-423-5p* and *miR-1306-5p*) and one down-regulated miRNA (*miR-378a-3p*) for validation in an independent HCC cohort (*n* = 46). RT-qPCR was conducted with the SYBR Green PCR Kit (TaKaRa, Beijing, China) using GAPDH as an internal control for the lncRNAs. The miRNA expression levels were normalized to the U6 spliceosomal RNA (U6). The lncRNA and miRNA expression levels are represented as fold changes using the $2\varDelta \varDelta {Ct}$. The primers were listed in Table S3 (Additional file 1).

### Validation of the role of *MEG3* by its overexpression *in vitro*

Human liver cancer cell line HepG2 and human liver cell line LO2 were cultured in Dulbecco’s modified Eagle’s medium (DMEM) containing 10% fetal bovine serum (FBS), 2 mM glutamine, 100 mg/ml streptomycin and 100 U/ml penicillin under a humidified atmosphere with 5% CO_2_ at 37 °C. The cell culture medium was changed every two days, and cells were passaged at 80–90% confluence with 0.25% trypsin (with 0.1% EDTA). The reconstructed isoform of *MEG3* by cufflinks was aligned to the human genome, and thereby we synthesized the sequence of the transcript of *MEG3* (GenBank accession number: MH929320) and cloned into pcDNA3.1(+) vectors. PcDNA3.1(+)-*MEG3* plasmids were transiently transfected into both HepG2 and LO2 cells using Lipofectamine 2000 transfection reagent (Invitrogen Life Technologies, Shanghai China). A group of cells was transfected with empty vector pcDNA3.1(+) as negative controls. Cells were recycled 48 h after transfection. Both *MEG3* transfected and control samples were conducted for transcriptome sequencing, including mRNA, lncRNA, and miRNA, at the Beijing Genome Institute (Beijing, China), as described previously. The original data for mRNA and miRNA in four samples of two cell lines were shown in Table S4 (Additional file 1). We randomly partitioned the original data for each sample into three subsets for statistical replication by down-sampling. DE analyses were performed using the protocol described above.

## Results

### RNA-seq identified differentially expressed miRNAs and lncRNAs

**Figure 1:**
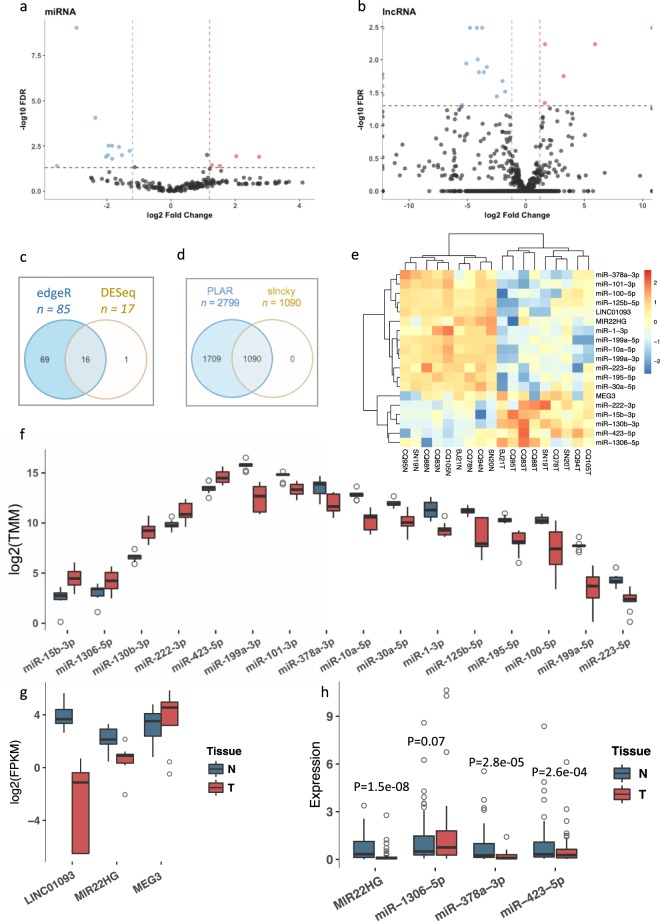
DE ncRNAs. A volcano plot of miRNAs (**a**) and lncRNAs (**b**). (**c**) Significantly DE miRNAs identified using two algorithms (edgeR and DESeq). (**d**) Filtration of lncRNAs using PLAR and slncky. (**e**) Heatmap of significantly DE ncRNAs. A boxplot showed the expression of the DE miRNAs (**f**) and lncRNAs (**g**). Each ncRNA contains two boxes with different colors (dark sky blue: normal and Indian red: tumor). (**h**) RT-qPCR results of selected DE ncRNAs (dark sky blue: normal and Indian red: tumor).

A total of 28 million raw miRNA sequencing reads were generated (Additional file 1, Fig. S3a), and approximately 87% were mapped to the reference genome with the length of 18–25 nt (36% with 22 nt) (Additional file 1, Fig. S3b). We detected 295 unique hairpins and 203 mature miRNAs in all samples (Additional file 1, Table S5); these miRNAs were further analyzed if they were identified in ≥50% of samples in each tissue and the number of uniquely mapped reads was >5. A total of 203 miRNAs were identified (Fig. 1a), which 85 miRNAs (identified by edgeR) were significantly aberrantly expressed. Combined with DESeq, 16 miRNAs (${\rm{|}}{\log }2({FC}){\rm{|}}$ >1.2 and p-adjusted value <0.05) (Table 1; Fig. 1c) were selected for further analyses. Overall, there were 11 down- and five up-regulated miRNAs with a wide range of FCs (e.g., −3.55 of ${\rm{|}}{\log }2({FC}){\rm{|}}$ for *miR-199a-5p* and 2.74 for *miR-130b-3p*) (Fig. 1c,f). The majority of DE miRNAs were significantly associated with HCC (Additional file 1, Table S6) according to the miRWalk and Ingenuity Pathway Analysis (IPA), although four miRNAs (*miR-223-5p, miR-378a-3p, miR-423-5p*, and *miR-15b-3p*) lacked experimentally validated evidence. We selected *miR-1306-5p, miR-378a-3p*, and *miR-423-5p* for further validation; the RT-qPCR results supported *miR-378a-3p* and *miR-423-5p*, with *p* = 2.8e-05 and 2.6e-04, respectively (Fig. 1h).

**Table 1:** Identification of significantly DE miRNAs.

miRNA	Chr. Location	log2FC	Pval	Padj	Family	Cluster
*miR-199a-3p*	19p13.2/1q24.3	−2.94	0e + 00	0e + 00	mir − 199	mir-199a-2/mir-3120/mir-214
*miR-10a-5p*	17q21.32	−2.36	8e − 07	8e − 05	mir − 10	NA
*miR-125b-5p*	11q24.1/21q21.1	−1.84	5e − 05	3e − 03	mir − 10	NA
*miR-223-5p*	Xq12	−1.95	6e − 05	3e − 03	mir − 223	NA
*miR-378a-3p*	5q32	−1.62	8e − 05	3e − 03	mir − 378	NA
*miR-101-3p*	1p31.3/9p24.1	−1.59	1e − 04	5e − 03	mir − 101	mir-101-1/mir-3671
*miR-423-5p*	17q11.2	2.17	3e − 04	9e − 03	mir − 423	mir-423/mir-3184
*miR-1-3p*	18q11.2/20q13.33	−1.96	4e − 04	1e − 02	mir − 1	mir-1-2/mir-133a-1
*miR-30a-5p*	6q13	−1.53	4e − 04	1e − 02	mir − 30	NA
*miR-15b-3p*	3q25.33	2.03	5e − 04	1e − 02	mir − 15	mir-15b/mir-16-2
*miR-195-5p*	17p13.1	−2.02	7e − 04	1e − 02	mir − 15	mir-497/mir-195
*miR-130b-3p*	22q11.21	2.74	7e − 04	1e − 02	mir − 130	mir-301b/mir-130b
*miR-100-5p*	11q24.1	−1.83	1e − 03	1e − 02	mir − 10	mir-100/let-7a-2
*miR-1306-5p*	22q11.21	1.57	2e − 03	3e − 02	mir − 1306	mir-3618/mir-1306
*miR-222-3p*	Xp11.3	1.52	3e − 03	4e − 02	mir − 221	mir-222/mir-221
*miR-199a-5p*	19p13.2/1q24.3	−3.55	3e − 03	4e − 02	mir − 199	mir-199a-2/mir-3120/mir-214

A total of 358.3 million reads were obtained for the lncRNAs, of which 92% mapped to the reference genome including different sequence length covering exons (Fig. S3c). Similar to the miRNA results, we retained lncRNAs that were expressed in at least 50% of the samples for each tissue with expression levels > 1 FPKM^39^. Both PLAR and slncky identified 1,090 plausible lncRNAs (Fig. 1b,d,e), which were classified into five categories (Additional file 1, Fig. S3d). A total of 24 potentially DE lncRNAs (14 novel and 10 known) were identified (${\rm{|}}{\log }2({FC}){\rm{|}}$ > 1.5 and *p-*adjusted value < 0.05). However, after manual review for the potentially DE lncRNAs based on annotations from multiple databases including Ensembl, UCSC, GeneCards, HGNC, LncRNADisease, Lnc2Cancer, and MNDR, we excluded ‘pseudogenes’ and remained three lncRNAs (*MEG3*, *MIR22HG*, and *LINC01093*) that were consistently annotated to be as ‘true’ lncRNAs (Table 2; Fig. 1g). *MEG3* and *MIR22HG* (validated with *p* = 1.5e-08) are known HCC-associated lncRNAs^40^. A previous study identified up-regulation of *LINC01093* expression in HCV and alcohol-associated HCC^41^), demonstrating its tumor-suppressing effect. However*, LINC01093* was down-regulated in our HCC cohort (Fig. 1g). The use of a combination of multiple approaches to identify concordance may strengthen the reliability.

**Table 2:** Identification of significantly DE lncRNAs.

lncRNA	Class	Chr. Location	Strand	log2FC	Pval	Padj
*MEG3*	Intergenic	14:101292444–101327360	+	2.66	5e − 05	0.003
*LINC01093*	Intergenic	4:185814153–185820615	−	−5.92	2e − 03	0.040
*MIR22HG*	mir_host_exon	17:1614797–1619566	−	−1.62	1e − 04	0.005

### Target collection illustrated potential interactions between non-coding RNAs

**Figure 2:**
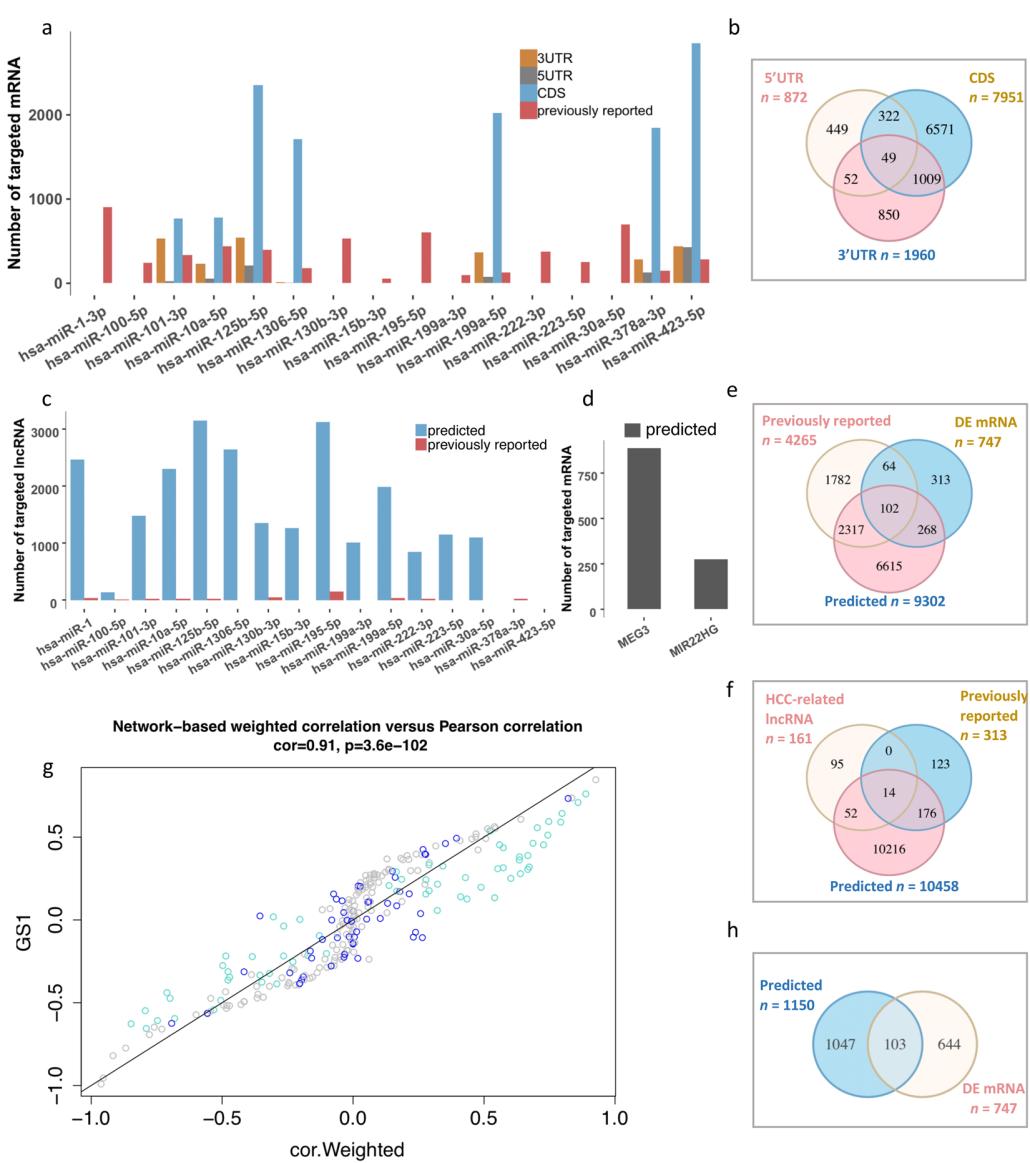
Interactions between miRNAs, lncRNAs, and mRNAs. (**a**) The number of miRNA-mRNA targets based on different sequence regions in both predicted and previously reported miRNA-targeted mRNAs. The 3′-UTR, 5′-UTR, and CDS were the regions of the targeted mRNAs that were used for the predictions. (**b**) A Venn plot of the miRNA-mRNA target predictions based on different regions. (**c**) A summary of the miRNA-lncRNA targets for both the predicted and previously reported interactions. (**d**) A summary of the lncRNA-mRNA targets for both the predicted and previously reported miRNA-targeted lncRNAs. Venn plots for the interactions of significantly DE miRNAs and mRNAs (**e**), miRNAs and lncRNAs (**f**), lncRNAs and mRNAs (**h**). and (**g**). A co-expression network of the MLMI based on weighted Pearson’s correlations (GS1: average gene significance; cyan: module 1; blue: module 2; and gray: non-modules).

The regulatory roles of the DE ncRNAs for their target mRNAs were investigated. First, 4,265 (31.4%) of the 9,302-potentially miRNA-targeted mRNAs predicted *in silico* were previously reported (miRTarBase) (Fig. 2a–c). *miR-125b-5p* and *miR-423-5p* exhibited larger numbers of consensus targets (457 and 340, respectively). Several miRNAs (e.g., *miR-100-5p* and *miR-130b-3p*) exhibited previously reported targets only due to the strict filtration criteria. A total of 166 of the reported mRNA targets were identified in the same cohort in our previous study (747 DE mRNAs, including 334 up-regulated and 413 down-regulated)^11^ (Fig. 2e). Specifically, *miR-1-3p* and *miR-30a-5p* interacted with approximately half of these mRNAs (Additional file 1, Fig. S4), which indicated its predominant role for these two miRNAs. We also noted that multiple miRNA-targeted mRNAs were genes associated with cancer (e.g., *TP53, PTEN, STAT3, VEGFA, CCND1*, and *CDK6*)^42^. Additionally, two lncRNAs (*MEG3* and *MIR22HG*) targeted 103 DE mRNAs (Fig. 2d,h; Additional file 1, Fig. S5). In contrast to the miRNA-targeted mRNAs, 20 DE mRNAs were targeted by both DE miRNAs and lncRNAs, including *NFAC, JUN*, and *EGR2*.

Next, we identified potential miRNA-targeted lncRNAs that played major roles as endogenous miRNA sponges^9^. Only a small proportion of the predicted interactions were previously reported (313 out of 10,458) (Fig. 2c,f). We also identified 243 miRNA-lncRNA targeting edges contributed by 66 (of 161) HCC-lncRNAs and 14 miRNAs (Additional file 1, Fig. S6). A DE miRNA targets multiple lncRNAs (e.g., *miR-195-5p* targets 30 lncRNAs), and a given lncRNA can be targeted by multiple miRNAs (e.g., *HOTAIR*, *MEG3* and *MIR22HG* interacted with ≥6 miRNAs, and *LINC01093* was targeted by multiple DE miRNAs) (Additional file 2). On average, a miRNA targeted approximately 20 HCC-associated lncRNAs, and a lncRNA was targeted by ≥2 miRNAs, indicating complex regulatory functions among these ncRNAs. Additionally, these ncRNAs may act in a coordinated manner, as exhibited with the ‘sponge’ function^9^. The DE miRNAs targeted several well-known HCC-associated lncRNAs, including *H19, HOTAIR*, and *HULC*.

### The MLMI network showed reciprocal interactions

**Figure 3:**
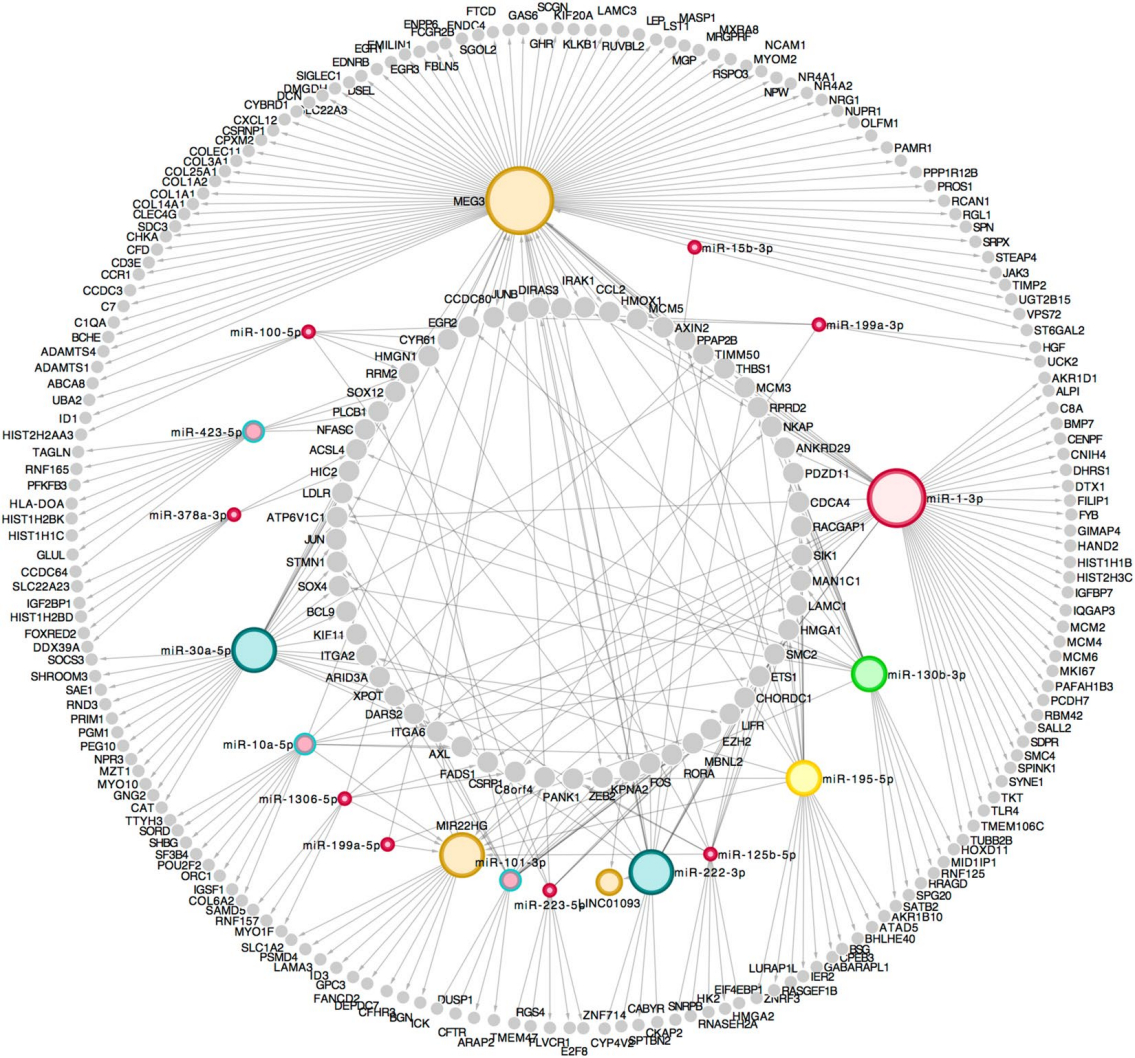
A MLMI network of significantly DE RNAs. Different colors indicate different RNA molecules (gray: mRNAs and colorful dots: ncRNAs). The outer circle includes mRNAs targeted by a single ncRNA, the middle circle is miRNAs and lncRNAs (three dots in khaki), and the inner circle is mRNAs targeted by multiple ncRNAs.

We constructed a co-regulatory relationship network of the RNAs suspected to underlie hepatocarcinogenesis based on their multi-reciprocal interactions. A three-dimensional mRNA-lncRNA-miRNA (MLMI) network, including 253 DE mRNAs, 16 DE miRNAs and three DE lncRNAs, was constructed based on potential interactions between any two of three molecules (Additional file. 2). Complex reciprocal regulation of different RNAs indicated very close interactions, as assessed by both the linkage (line-based, each edge with *p* < 0.05) and co-expression (network-based) approaches (*r* = 0.91, *p* = 3.6e-102, Figs 2g and 3). Notably, a larger number of edges was shared by *MEG3, miR-1-3p*, and *miR-30a-5p*, and a significant correlation of expression was observed for *miR-1306-5p/miR-195-5p-MEG3-JAK3/COL1A1* (Pearson correlation: *r* = 0.87 and *p* < 0.01, line-based). *MEG3* is a core gene in the proposed MLMI network that plays a dominant role in both the regulation of multiple mRNAs and sponging of several miRNAs. *MEG3* has been shown to be involved in the epigenetic regulation of the epithelial-mesenchymal transition (EMT) in lung cancer cell lines^43^. *MEG3* significantly targeted 11 of the 33 DE genes related to the EMT (e.g., *COL1A2, CYR61*, and *FBLN5*), which suggested that its role in the EMT was associated with carcinogenesis. To investigate the MLMI network in another dataset, we characterized significantly DE mRNAs and miRNA in TCGA HCC cohort, and lncRNAs identified previously^44^. Using the same workflow, an enormous mRNA-lncRNA-miRNA network was predicted underlying TCGA 377 HCC patients (Additional file 3).

**Figure 4:**
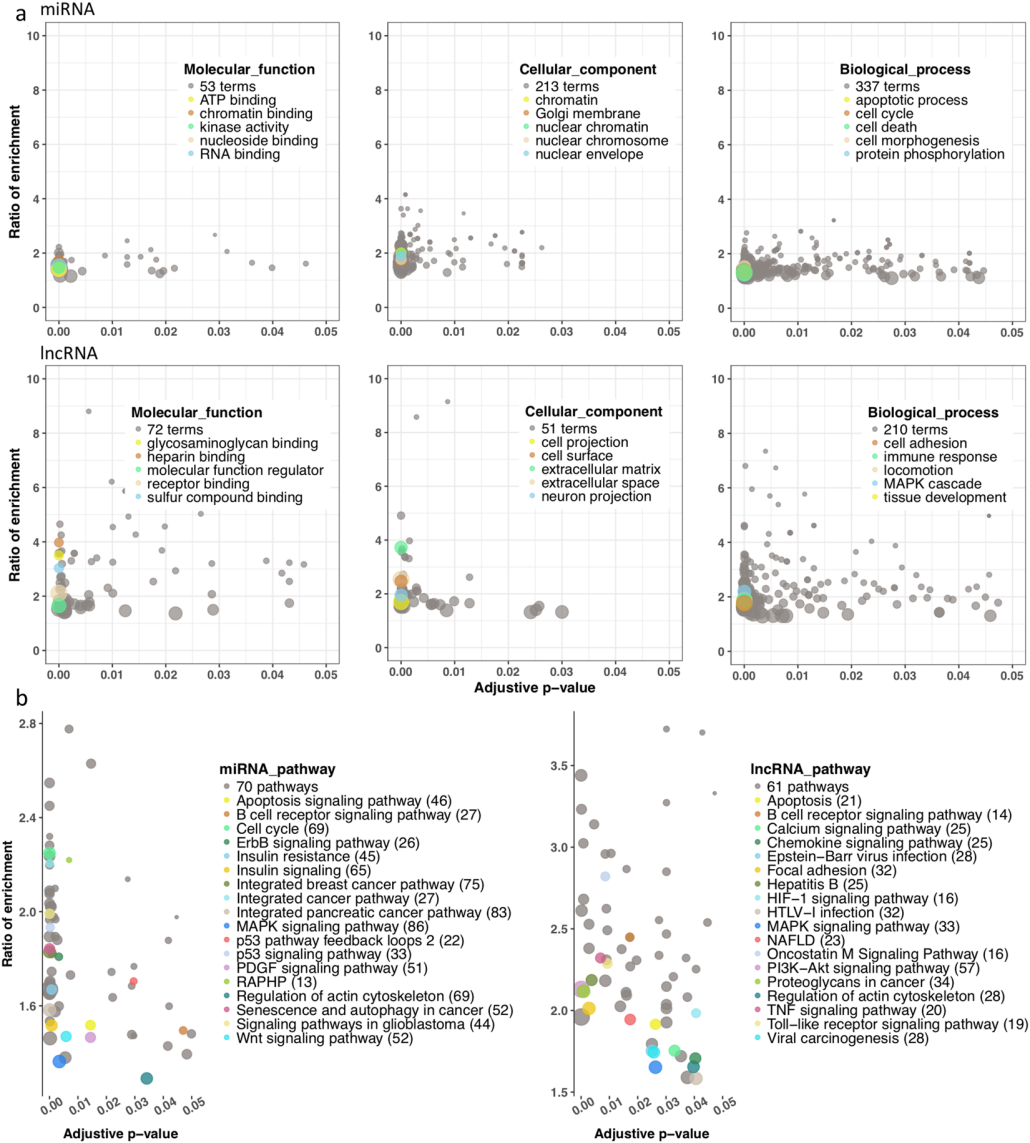
Analyses of functional pathway for ncRNAs. (**a**) Gene ontology (GO) analysis of the cellular localization, biological processes and molecular functions of the miRNAs and lncRNAs. Each point represents a GO term that is highly enriched for the non-coding RNAs, and the total number of each GO class is listed in the legend. The most significant terms (*p* < 10^−15^) are presented. Points in gray indicate significant enrichment of GO terms (*p* < 0.05). (**b**) Functional pathway analyses of miRNAs and lncRNAs using KEGG, Panther, and Wiki. Each point represents a pathway, and points with different colors represent the HCC-associated pathways listed in the legend. The numbers in round brackets represent the number of genes that operate in that pathway predominantly from our input gene list. The total number of significantly enriched pathways obtained from all three databases is shown in the legend.

Gene ontology (GO) analysis identified 2,619 terms for miRNAs and 1,455 terms for lncRNAs. The most significant biological processes (*p* < 0.01 and FDR < 0.05) for both miRNAs and lncRNAs were the cell cycle, apoptosis, chromatin binding and RNA binding (Fig. 4a). We also noted roles for the MAPK cascade and heparin binding, which were consistent with the pathway analysis. Functional pathway enrichment analyses of the miRNAs and lncRNAs identified a total of 975 pathways and revealed their roles in HCC development. For the mRNAs regulated by the 16 DE miRNAs, 76 pathways were significantly enriched, and 18 validated pathways were implicated in HCC (e.g., hepatitis B, NAFLD and Wnt signaling) (Fig. 4b). For the mRNAs targeted by the three lncRNAs, 62 pathways were significantly enriched, and 18 validated pathways were implicated in HCC (Fig. 4b). Intriguingly, most (15/18) of the HCC-related pathways were consistent, which suggested a co-regulatory mechanism. The most significant pathways were apoptosis, MAPK, and p53 signaling, which highlighted the essential roles of both miRNAs and lncRNAs in HCC development. *miR-199a-3p* was not significantly enriched in our cohort due to limited expression, but this miRNA was reported to regulate the mTOR pathway in human hepatocarcinoma cells^45^.

### Validation of *MEG3*-targeted miRNAs and mRNAs by its overexpression *in vitro*

**Figure 5:**
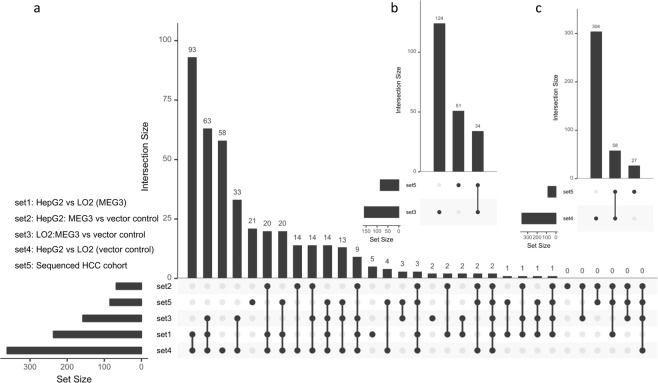
The role of *MEG3* validated by its overexpression *in vitro*. (**a**) The intersection of DE miRNAs identified *in vitro* (HepG_2_ and LO_2_ cell lines) and the sequenced HCC cohorts. (**b**) DE miRNAs identified in the sequenced HCC that was validated *in vitro*. (**c**) DE miRNAs identified in LO_2_ with overexpression of *MEG3*.

Sequence analysis revealed that the reconstructed *MEG3* is a potentially novel isoform. Given its predominant role in the MLMI network, we investigated how the overexpression of *MEG3* altered the expression profiles in a normal hepatic cell line (LO2) and one of the hepatocellular cell lines (HepG2), since the expression of *MEG3* was increased in the cancer tissues (${\log }2({FC})$ = 2.66; p = 0.003) in our sequenced cohort. Compared with the vector control, we identified 158 DE miRNAs and 101 mRNAs in the *MEG3* transiently-transfected LO2 cell line (Figs 5a,c and S7a). Of the 85 DE miRNAs identified in our HCC cohort, 34 were noted to be significantly differentially expressed in the LO2 cell line (Figs 5a,c and S7a,c). We then investigated the changes of expression for *MEG3* targeted miRNAs and mRNAs. Of the 45 DE miRNAs that was predicted to be sponged significantly by *MEG3*, 19 were validated to be up- or down-regulated in the LO2 cell line (Figs 5b; S7c). Our results showed that the overexpression of *MEG3* can alter the expression of a set of miRNAs and mRNAs in non-HCC-background environment. We then investigated the changes of expression profiles in the hepatocellular carcinoma cell line HepG2 in the setting of overexpression of *MEG3*. When compared with the vector controls, 68 DE miRNAs (15 up- and 53 down-regulated) and 50 DE mRNAs (25 up- and 25 down-regulated) were identified in the HepG2 cell line (Figs 5b,c and S7b,c), of which 21 miRNAs were potentially interacted with *MEG3* (e.g., *miR-195-5p*). Therefore, overexpression of *MEG3* in the HepG2 cell line could disturb the expression profiles, especially in miRNAs or mRNAs that was interacted with *MEG3*. Taken together, our findings validated the regulation function of an isoform of *MEG3 in vitro* and a proportion of potential targets of *MEG3* characterized in the MLMI network.

### Clinical implications of ncRNAs in the MLMI network in survival

**Figure 6:**
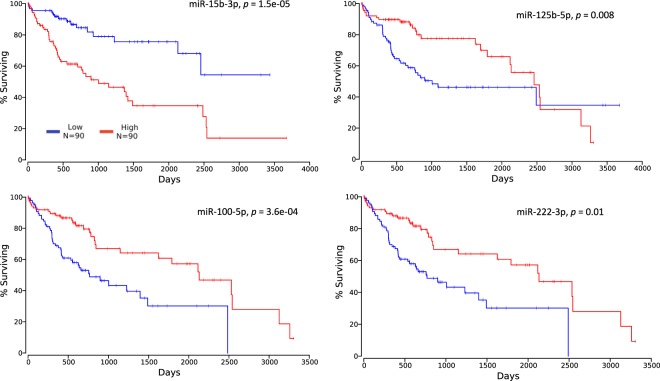
Survival analysis of TCGA HCC cohort based on the DE ncRNAs (red line: patients with a higher expression levels (upper 25%, *n* = 90); blue line: patients with a lower expression levels (lower 25%, *n* = 90).

We investigated whether these DE ncRNAs could be used as potential biomarkers for the prognosis of HCC. The survival analysis showed that, of the 16 miRNAs shown in the MLMI network, the levels of *miR-15b-3p, miR-100-5p, miR-125b-5p*, and *miR-222-3p* were significantly associated with the probability of survival (*p* < 0.01, Fig. 6). Higher *miR-15b-3p* expression indicated poor survival in HCC patients (*p* = 1.5e-05), and the expression levels of the remaining three miRNAs (*miR-100-5p, miR-125b-5p*, and *miR-222-3p*) were positively correlated with the survival probability (*p* = 3.6e-04, 0.8e-03 and 0.01, respectively). These results suggest that a subset of miRNAs characterized in the MLMI network can be used as prognostic biomarkers for HCC.

## Discussion

In the present study, we reported a comprehensive characterization of ncRNA expression profiles in HCC, a novel *MEG3* isoform with potential oncogenic function *in vitro*, and the construction of a *MEG3*-predominated MLMI network using significantly DE lncRNAs, miRNAs and mRNA to reveal multiple interactions among these molecules during post-transcriptional regulatory processing.

Abundantly expressed miRNAs have been suggested to be more important than miRNAs that are expressed at relatively low levels since a minimum threshold of expression must be reached to repress the expression of their targets^46^. For example, increased *miR-423-5p* expression (Fig. 1f) regulates the G1/S transition via targeting p21Cip1/Waf1, which promotes cell growth^47^), as an oncogenic event in HCC development. However, poorly expressed miRNAs may also play roles in carcinogenesis (e.g., down-regulated *miR-15b-3p* and *miR-223-5p* are relevant for HCC proliferation and metastasis, and *miR-199a/b-3p* inhibits HCC growth *in vitro* and *in vivo*^48^). In the present study, we showed for the first time that the down-regulated *miR-1306-5p* significantly targeted *MEG3* and *RNF157*, which promote cell proliferation and HCC migration^49^. Over-expressing *MEG3* into the LO2 and HepG2 replicated partial tissue-based expression profile, suggesting the consistency and heterogeneity between tissue- and cell-sample environment. The expression profile change under over-expression the novel isoform of *MEG3* validated and highlighted its important post-regulating function.

The ncRNAs that were significantly enriched in the HCC-related signaling pathways (e.g., MAPK) also played important roles in the immune response (Fig. 4b). For example*, CD3E* is an immune marker gene that is targeted by *MEG3* with a co-expression pattern. Another enriched pathway in our ncRNA molecules was the virus-involved pathways (especially HBV infection), suggesting that ncRNAs also participate in the HBV etiology the leads to HCC. Zheng *et al*.^50^ investigated the infiltrating T cells in liver cancer and highlighted the importance of the immune phenotype in HCC. We also identified an enrichment of B and T cell pathways in our cohort (Fig. 4).

Several studies have investigated direct interactions between RNAs in HCC^10^, including miRNA-mRNA^51^, lncRNA-mRNA^2^, and miRNA-lncRNA^52^ interactions. However, the complex regulation by multiple ncRNAs has not been examined previously. We constructed a MLMI network in HCC that shed new light on the roles of different RNA molecules in hepatocarcinogenesis. Most of the dysregulated modules in the network were mRNAs regulated by down-regulated miRNAs, which indicated a negative regulatory role for the miRNAs. However, a subset of the up-regulated mRNAs (*n* = 22) were targeted by both the up- and down-regulated miRNAs but were dominated by the up-regulated miRNAs, which suggested an antagonistic mechanism for post-transcription regulation. Second, as posited in the Steiner tree graph theory concept (i.e., the shortest path is the preference for molecular interactions in network-based regulation), our MLMI network showed that three-molecule pathways also played an essential role in HCC development. Zheng *et al*.^53^ validated the interaction path of *miR-1231-LINC00673-PTPN11*, which might maintain cell homeostasis and confer susceptibility to pancreatic cancer. Due to significant tumor heterogeneity, the MLMI network predicted in our sequenced samples was not fully replicated in TCGA HCC datasets. However, approximately 13 million MLMI ‘trios’ were constructed underlying TCGA HCC suggested that the MLMI networks is ubiquitous, which provide important implications for the function of ncRNAs underlying hepatocarcinogenesis. However, statistical evidence for ncRNAs in diseases requires mechanistic explanations from the perspective of interaction modules rather than single genes.

The present study has several limitations. First, as explained in our previous work^11^, the power to detect DE non-coding RNAs was limited due to the small sample size; for example, several well-known HCC-related DE miRNAs (*H19*, *HULC*, *miR-214-3p*, and *miR-122*) were not identified. In addition, a number of genes that were differently expressed in another dataset that may affect tumor progression or prognosis due to tumor heterogeneity. Second, our analysis excluded genes expressed at extremely low levels. Expression variability may be disturbed by loss-of-function mutations, transcriptional noise, and tumor heterogeneity. Therefore, the potential functions of ncRNAs with low expression levels should be interpreted with caution. Consequently, the established MLMI network is restricted to our cohort, and the inclusion and coverage of the network are limited. Notably, a significant interaction between different RNA molecules survived our harsh selection criterion. Third, the MLMI network is marked with sample uniqueness and is not generally applicable to other datasets. Combining more datasets may effectively elucidate the underlying regulatory relationships. However, at least a proportion of potential targets of *MEG3* characterized in the MLMI network was validated *in vitro*. Finally, *in vivo* experiments are needed further for validation of multiple interactions of miRNA, lncRNA and mRNAs.

In conclusion, we characterized DE miRNAs and lncRNAs between HCC and adjacent non-cancer tissues and constructed a MLMI network based on validated reciprocal interactions between ncRNAs and mRNAs in HCC, as well as identified a novel isoform of *MEG3* for the first time. Our work suggests that complex interactions among miRNAs, lncRNAs, and mRNAs may underlie hepatocarcinogenesis^54^.

## Supplementary information

Additional file 1

Additional file 2

Additional file 3

## Data Availability

These sequence data have been submitted to the BioSample database (hosted by the NCBI) (http://www.ncbi.nlm.nih.gov/biosample), under accession number PRJNA315318. The shell scripts, R codes, and related data/files for the study were deposited under the Google drive space (https://drive.google.com/drive/u/1/folders/1Wl6jzfdL4UKeCsDqfrqy5flp77w49iYc).
