# Supplementary material for: Transcriptomic Analysis of mRNA-lncRNA-miRNA Interactions in Hepatocellular Carcinoma
Source: Sci Rep. 2019 Nov 6;9:16096. doi: 10.1038/s41598-019-52559-x (PMC6834564; doi:10.1038/s41598-019-52559-x)
Supplement: Supplementary file 1 — Additional file 1 [file 41598_2019_52559_MOESM1_ESM.docx]

### Title page

Transcriptomic Analysis of mRNA-lncRNA-miRNA Interactions in Hepatocellular Carcinoma

Xia Tang^1^, Delong Feng^2^, Min Li^3^, Jinxue Zhou^4^, Xiaoyuan Li^5^, Dachun Zhao^6^, Bingtao Hao^2,7^, Dewei Li^8^, and Keyue Ding^1,9,^*

^1^ Key Laboratory of Molecular Biology for Infectious Diseases (Ministry of Education), Institute for Viral Hepatitis, Department of Infectious Diseases, The Second Affiliated Hospital, Chongqing Medical University, Chongqing, P.R. China

^2.^Cancer Research Institute, Southern Medical University, Guangzhou, Guangdong, P.R. China

^3^ Department of Hepatobiliary Surgery, Suining Central Hospital, Suining, Sichuan Province, P.R. China
^4^ Department of Hepatobiliary Surgery, Henan Tumor Hospital, Zhengzhou, Henan, P.R. China
^5^ Department of Medical Oncology, Peking Union Medical College Hospital, Peking Union Medical College and Chinese Academy of Medical Sciences, Beijing, P.R. China

^6^ Department of Pathology, Peking Union Medical College Hospital, Peking Union Medical College and Chinese Academy of Medical Sciences, Beijing, P.R. China

^7^ Henan Medical Genetics Institute, People's Hospital of Henan University, Zhengzhou, Henan, P.R. China
^8^ Department of Hepatobiliary Surgery, The First Affiliated Hospital of Chongqing Medical University, Chongqing, P.R. China

^9^ Department of Bioinformatics, Basic Medical College, Chongqing Medical University, Chongqing, P.R. China

Keyue Ding, Ph.D.
1# Road Yixueyuan, Yuzhong District, Chongqing, 400010 P.R. China
Phone: +86 23 6389 2759
E-mail: [ding.keyue@gmail.com](mailto:ding.keyue@gmail.com)

### Supporting Information

 Fig. S1. A bioinformatics pipeline for analyzing miRNA and lncRNA. (A). Identification of the known miRNAs with DE profiles. The correlation between miRNAs and HCC-related mRNAs are elucidated using target prediction. (B). Identification of lncRNAs with multiple filters. A combination of significantly HCC-related miRNAs, these targeted mRNAs and lncRNAs highly connected with HCC, was applied to construct a mRNA-lncRNA-miRNA (MLMI) network underlying hepatocarcinogenesis. Blue box: the elements used as nodes and edges in MLMI network.

 Fig. S2. Targets collection. The left 14 algorithms were used to predict targets between any two of three RNA molecules. Specifically, miRNA-mRNA targets were predicted via the first 12 algorithms, including 3’UTR (text in black), 5’UTR (blue text with yellow border) and CDS (Italic text in blue) of targeted mRNA. Four stared algorithms were used to predict targets between miRNA and lncRNA. lncRNA and mRNA interactions were investigated using the last two algorithms in green. Respectively, each interaction was validated correspondingly via the right three databases.


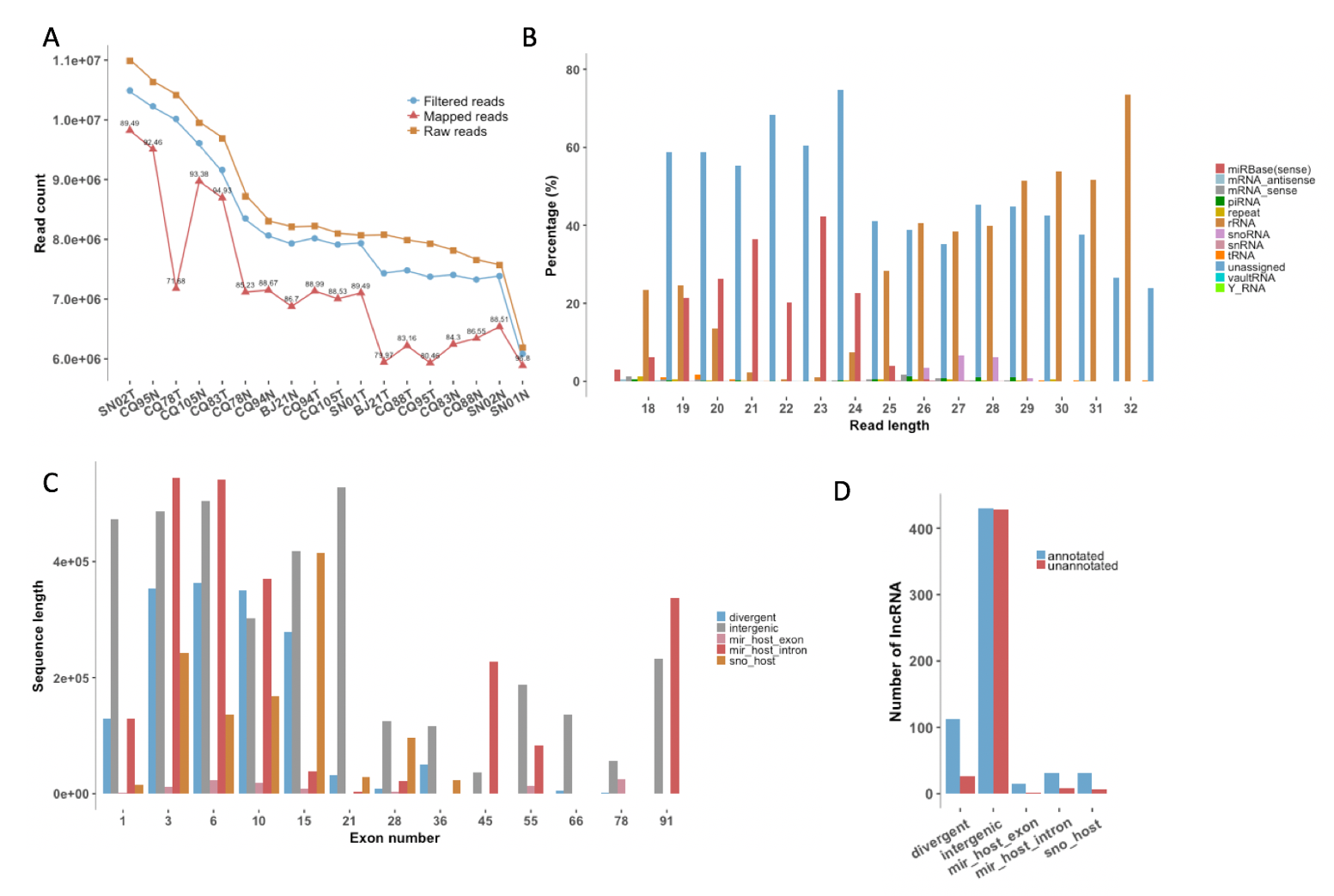
 Fig. S3. a summary of sequencing and mapping of ncRNAs. a. The filteration and mapping of raw sequencing reads for miRNAs using sRNAbench. Yellow: raw reads after removing adapters; Blue: reads after filtering; Pink: the count of mapped reads. The mapping rate (%) was shown. b. Sequence length distribution of different RNAs after alignment. Red: mature miRNAs aligned to the sense mature miRNA library in the miRBase. c. Genomics features of lncRNAs from exon number and sequence length. 'divergent': long divergent non-coding RNA; 'intergenic': long intergenic non-coding RNA; 'mir_host_exon': miRNA host gene lie in the exon; 'mir_host_intron': miRNA host gene lie in the intron; 'sno_host': small non-coding RNA host gene. d. The annotation of lncRNAs. Y-axis indicates the number of the identified lncRNAs. Sky blue: annotated; Indian red: unannotated.

 Fig. S4. A sub-network of miRNA-mRNA interactions. Nodes represent miRNAs with different sizes according to their contribution to interaction edges. Gray is DE mRNA, and the inner circle indicates mRNA targeted by multiple miRNAs, whereas these were regulated by a single miRNA form the outside circle.

 Fig. S5. A sub-network of lncRNA-mRNA interactions. Only two lncRNAs are observed to interacted with mRNAs via *trans*, and only *ITGA6* is targeted by both lncRNAs.

 Fig. S6. A sub-network of miRNA-lncRNA interactions. Nodes represent miRNAs with different sizes according to their contribution of interaction edges. Gray is HCC-related lncRNAs


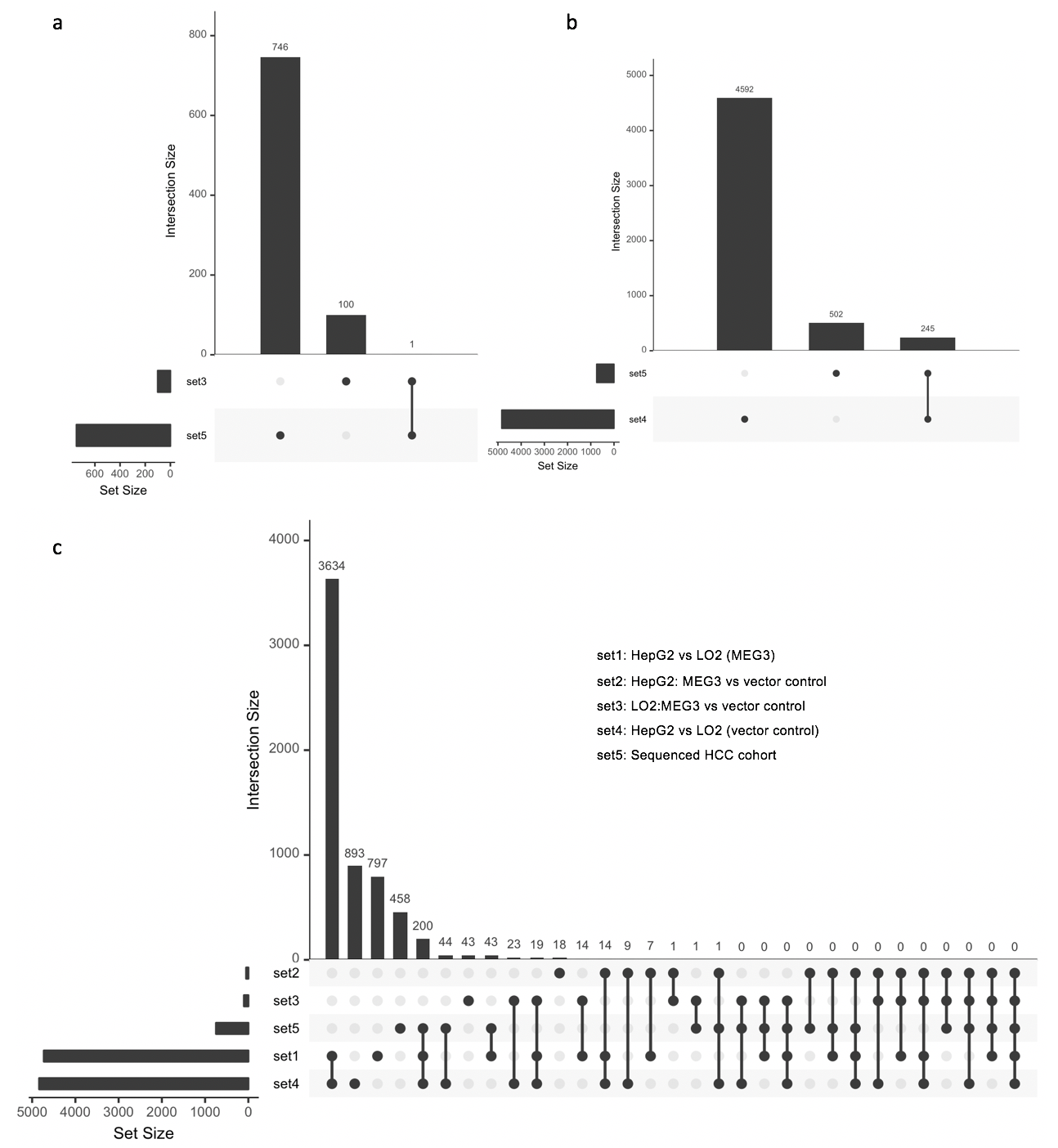


Fig. S7. The role of *MEG3* validated by its overexpression *in vitro*. a. DE mRNAs identified in the sequenced HCC that was validated *in vitro*. b. DE mRNAs identified in LO_2_ with overexpression of *MEG3*. c. The intersection of DE mRNAs identified *in vitro* (HepG_2_ and LO_2_ cell lines) and the sequenced HCC cohorts.

Table S1. A summary to clinical features of 46 HCC patients for RT-qPCR validation.

| ID | Sex | Age (y) | HBV DNA | HBsAg | HBeAg | AFP1(ng/ml) |
| --- | --- | --- | --- | --- | --- | --- |
| CQ100 | Male | 61 | 3.51E+05 | + | + | 86638 |
| CQ133 | Male | 30 | 1.48E+05 | + | + | >484000 |
| CQ134 | Male | 41 | NA | + | + | 467.88 |
| CQ136 | Male | 64 | 3.06E+04 | + | - | 6.42 |
| CQ137 | Male | 32 | <1.0E+03 | + | - | 1.04 |
| CQ138 | Male | 40 | 3.42E+04 | + | - | 277.2 |
| CQ141 | Male | 55 | <1.0E+03 | + | - | 200 |
| CQ143 | Female | 52 | 5.51E+05 | + | + | 75620 |
| CQ148 | Male | 49 | NA | + | - | 6837 |
| CQ149 | Male | 48 | <1.0E+03 | + | - | 1.37 |
| CQ154 | Male | 50 | 4.89E+03 | + | - | 1.45 |
| CQ155 | Male | 55 | 6.85E+03 | + | - | 32.46 |
| CQ157 | Male | 35 | <1.0E+03 | + | + | 1144 |
| CQ158 | Male | 36 | <1.0E+03 | + | - | 45.68 |
| CQ161 | Male | 57 | 1.61E+05 | + | + | 3421 |
| CQ163 | Male | 52 | 2.91E+05 | + | - | 97.65 |
| CQ175 | Male | 27 | <1.0E+03 | + | - | 232.51 |
| CQ176 | Female | 38 | <1.0E+03 | + | - | 179.44 |
| CQ177 | Male | 56 | 7.82E+03 | + | - | 359.6 |
| CQ178 | Male | 68 | 1.25E+03 | + | - | 5.86 |
| CQ179 | Male | 44 | 2.98E+07 | + | + | NA |
| CQ183 | Male | 56 | 3.98E+05 | + | - | 7.15 |
| CQ185 | Male | 43 | 1.40E+04 | + | - | 3.74 |
| CQ186 | Male | 49 | 2.36E+04 | + | - | 1210 |
| CQ187 | Male | 58 | NA | + | - | 15.81 |
| CQ190 | Male | 50 | <1.0E+03 | + | - | 25.45 |
| CQ192 | Male | 64 | <1.0E+03 | - | - | 30.65 |
| CQ193 | Male | 77 | <1.0E+03 | - | - | 3.31 |
| CQ195 | Male | 66 | <1.0E+03 | - | - | 3.31 |
| CQ76 | Male | 58 | <1.0E+03 | + | - | 8.19 |
| CQ80 | Male | 31 | NA | - | - | 1.85 |
| CQ89 | Male | 62 | <1.0E+03 | - | - | 5.32 |
| CQ91 | Male | 38 | 2.08E+03 | + | + | 848.6 |
| CQ93 | Female | 40 | <1.0E+03 | + | + | 143.4 |
| CQ96 | Male | 49 | 4.40E+04 | + | - | 2.1 |
| CQ99 | Male | 58 | 4.60E+06 | + | + | 2.49 |
| HN07 | Male | 66 | 2.92E+04 | + | + | >1210 |
| HN30 | Male | 51 | <5.00E+02 | + | + | 2.46 |
| SN15 | Male | 62 | 1.15E+05 | + | - | 1210 |
| SN16 | Male | 43 | <5.00E+02 | + | - | 5.29 |
| SN23 | Male | 49 | NA | + | - | 8.22 |
| SN27 | Female | 41 | 1.50E+03 | + | - | 868.4 |
| SN28 | Male | 61 | 9.24E+05 | + | - | 484.8 |
| SN29 | Male | 48 | <5.00E+02 | + | - | 5.09 |
| SN30 | Male | 69 | <5.00E+02 | + | - | 89.98 |
| SN31 | Female | 50 | <5.00E+02 | + | - | 1210 |

Table S2. A summary of reported HCC-related lncRNAs.

| LncRNAs | | | |
| --- | --- | --- | --- |
| AF070632 | GLT1D1 | P6391 | SNHG11 |
| AF085935 | H19 | P6488 | SNHG12 |
| AFAP1-AS1 | HEIH | P700 | SNHG14 |
| AK021443 | HNF1A-AS1 | P8611 | SNHG15 |
| AK055007 | HOTAIR | P8725 | SNHG16 |
| AK056988 | HOTAIRM1 | P8860 | SNHG17 |
| AK093543 | HOTTIP | P9745 | SNHG18 |
| AK123790 | HOXA13 | PANDAR | SNHG20 |
| ANRIL | HULC | PAR5 | SNHG3 |
| AOC4P | ICR | PCAT-29 | SOX2OT |
| ATB | IGF2-AS | PCNA-AS1 | SPRY4-IT1 |
| AY129027 | KCNQ1OT1 | PFKFB1 | SRHC |
| BAIAP2-AS1 | LALR1 | PRAL | TCF7 |
| BC014579 | LET | PTENP1 | TCONS_00018278 |
| BC017743 | LINC00152 | PVT1 | TMEVPG1 |
| BC043430 | LINC00974 | RERT | TSPAN8 |
| C14orf132 | LINC01093 | ROR | TUC339 |
| CALCA | LINC01152 | RP1-102H19.7 | TUG1 |
| CCAT1 | LINC01419 | RP1-71H19.2 | UCA1 |
| CCAT2 | LOC105369388 | RP11-160H22.5 | UFC1 |
| CPS1-IT1 | LOC149086 | RP11-203H19.2 | ULK4P2 |
| CR613944 | MALAT1 | RP11-276H19.1 | URHC |
| CTB-167B5.2 | MEG3 | RP11-284H19.1 | VLDLR |
| CTC-344H19.4 | MIR22HG | RP11-304L19.5 | WRAP53 |
| CTD-2587H19.2 | MT1DP | RP11-343H19.1 | WT1-AS |
| CTD-2589H19.6 | MVIH | RP11-401P9.4 | XIST |
| CTD-3214H19.6 | NCRNA00173 | RP11-454H19.2 | XLOC_014172 |
| D16366 | NEAT1 | RP11-491H19.1 | Y3 |
| DANCR | NR_015378 | RP11-501G6.1 | ZEB1-AS1 |
| DBH-AS1 | NR_024284 | RP11-672F9.1 | ZFAS1 |
| DILC | NR_026591 | RP11-89H19.1 | aHIF |
| DQ786243 | NR_027151 | RP11-96H19.1 | hDREH |
| DREH | P14695 | RP4-794H19.1 | hPVT1 |
| EGFR-AS1 | P16984 | RP5-1014O16.1 | p21 |
| ENST00000395084 | P19780 | RP5-1185H19.2 | uc.338 |
| ENST00000501583 | P23099 | RP5-849H19.3 | uc001lsz |
| EVI1 | P24363 | SIRT1-AS | uc001ncr |
| FTX | P28210 | SLC25A27 | uc002mbe.2 |
| GAS5 | P33863 | SLC7A6 | uc002pyc |
| GAS5-AS1 | P4091 | SNHG1 | uc003yqb.1 |
|  |  |  | uc004bdv.3 |

Table S3. A summary of DE ncRNAs primer for experimentally validation.

| miRNA | Sense Primer | Antisense Primer |
| --- | --- | --- |
| miR-378a-3p | 5'-AACTGGACTTGGAGTCAGAAGGC-3' | - |
| miR-423-5p | 5'-ATGAGGGGCAGAGAGCGAGA-3' | - |
| miR-1306-5p | 5'-ACCACCTCCCCTGCAAACGT-3' | - |
| MIR22HG | 5'-CTGTATCTTGTCCTCCGCTTGTG-3' | 5'-TGTTGCAGCCTATGAGTCTATCCC-3' |

Table S4. Summary of original data for the MEG3 transfected LO2 and HepG2 RNA-seq.

| RNA-seq | LO2_control | LO2_MEG3 | HepG2_control | HepG2_MEG3 |
| --- | --- | --- | --- | --- |
| miRNA | 2.9G | 3.2G | 4.3G | 3.9G |
| mRNA | 12G | 12G | 12G | 12G |

Table S5. A summary of detected sense hairpins, isoforms, and mature miRNAs within each sample.

| Sample ID | miRNA | Hairpin | Isoform |
| --- | --- | --- | --- |
| BJ21B | 394 | 233 | 239 |
| BJ21C | 470 | 265 | 299 |
| CQ78B | 393 | 235 | 242 |
| CQ78C | 390 | 213 | 195 |
| CQ83B | 329 | 213 | 194 |
| CQ83C | 486 | 265 | 324 |
| CQ88B | 357 | 220 | 214 |
| CQ88C | 387 | 242 | 227 |
| CQ94B | 333 | 207 | 198 |
| CQ94C | 301 | 192 | 197 |
| CQ95B | 350 | 219 | 218 |
| CQ95C | 357 | 222 | 214 |
| CQ105B | 390 | 235 | 244 |
| CQ105C | 303 | 194 | 177 |
| SN19B | 331 | 209 | 195 |
| SN19C | 391 | 238 | 242 |
| SN20B | 344 | 213 | 200 |
| SN20C | 339 | 217 | 206 |

Table S6. A summary of proof for DE miRNAs under report with HCC-involved.

| miRNA | miRwalk | IPA | Reference |
| --- | --- | --- | --- |
| miR-199a-3p | + | + | [1–3] |
| miR-10a-5p | - | + | [4] |
| miR-125b-5p | + | - | [5] |
| miR-223-5p | - | - | Yes |
| miR-378a-3p | - | - | Yes |
| miR-101-3p | + | - | [6] |
| miR-423-5p | - | - | Yes |
| miR-1-3p | - | + | [4] |
| miR-30a-5p | - | + | [7] |
| miR-15b-3p | - | - | Yes |
| miR-195-5p | + | + | [1,7,8] |
| miR-130b-3p | - | + | [8] |
| miR-100-5p | - | + | [9] |
| miR-1306-5p | - | - | Yes |
| miR-222-3p | + | + | [10,11] |
| miR-199a-5p | + | + | [1,3] |

### Reference

1. Murakami Y, Yasuda T, Saigo K, Urashima T, Toyoda H, Okanoue T, et al. Comprehensive analysis of microRNA expression patterns in hepatocellular carcinoma and non-tumorous tissues*. Oncogene*. 25:2537–45 (2006)

2. Hou J, Lin L, Zhou W, Wang Z, Ding G, Dong Q, et al. Identification of miRNomes in human liver and hepatocellular carcinoma reveals miR-199a/b-3p as therapeutic target for hepatocellular carcinoma. *Cancer Cell.* 19:232–43 (2011)

3. Fornari F, Milazzo M, Chieco P, Negrini M, Calin GA, Grazi GL, et al. MiR-199a-3p regulates mTOR and c-Met to influence the doxorubicin sensitivity of human hepatocarcinoma cells. *Cancer Res.* 70:5184–93 (2010)

4. Sukata T, Sumida K, Kushida M, Ogata K, Miyata K, Yabushita S, et al. Circulating microRNAs, possible indicators of progress of rat hepatocarcinogenesis from early stages. *Toxicol Lett.* 200:46–52 (2011)

5. Liang L, Wong C-M, Ying Q, Fan DN-Y, Huang S, Ding J, et al. MicroRNA-125b suppressesed human liver cancer cell proliferation and metastasis by directly targeting oncogene LIN28B2. *Hepatology.* 52:1731–40 (2010)

6. Wei X, Xiang T, Ren G, Tan C, Liu R, Xu X, et al. miR-101 is down-regulated by the hepatitis B virus x protein and induces aberrant DNA methylation by targeting DNA methyltransferase 3A. *Cell Signal.* 1–32 (2012)

7. Huang Y-S, Dai Y, Yu X-F, Bao S-Y, Yin Y-B, Tang M, et al. Microarray analysis of microRNA expression in hepatocellular carcinoma and non-tumorous tissues without viral hepatitis. *J. Gastroenterol Hepatol.* 23:87–94 (2007)

8. Yang L, Ma Z, Wang D, Zhao W, Chen L, Wang G. MicroRNA-602 regulating tumor suppressive gene RASSF1A is over-expressed in hepatitis B virus-infected liver and hepatocellular carcinoma. *Cancer Biol. Ther.* 9:803–8 (2014)

9. Li D, Liu X, Lin L, Hou J, Li N, Wang C, et al. MicroRNA-99a inhibits hepatocellular carcinoma growth and correlates with prognosis of patients with hepatocellular carcinoma. *J. Biol. Chem.* 286:36677–85 (2011)

10. Wong QW-L, Ching AK-K, Chan AW-H, Choy K-W, To K-F, Lai PB-S, et al. MiR-222 overexpression confers cell migratory advantages in hepatocellular carcinoma through enhancing AKT signaling. *Clin. Cancer Res.* 16:867–75 (2010)

11. Garofalo M, Croce CM. microRNAs: Master Regulators as Potential Therapeutics in Cancer. *Annu. Rev. Pharmacol. Toxicol.* 51:25–43 (2011)
